# Supplementary material for: Relative Dose Intensity of Daratumumab, Lenalidomide, and Dexamethasone in Multiple Myeloma
Source: Cancers (Basel). 2025 Jan 30;17(3):470. doi: 10.3390/cancers17030470 (PMC11816031; doi:10.3390/cancers17030470)
Supplement: Supplementary file 1 [file cancers-17-00470-s001.zip › cancers-3366528-supplementary.pdf]

Supplementary Table S1. Patient Characteristics for high and low lenalidomide.

	**high RDI of LEN (n=54)**	**low RDI of LEN (n=57)**	***P* value**
Age			
Mean age	72 ± 5	75 ± 8	0.017
≥75year	17	37	<0.001
<75year	37	20	
Sex			
male	29	34	0.569
female	25	23	
ECOG performance status			
0,1	47	41	0.062
2,3,4	7	16	
Tyep of monoclonal protein			
IgG	20	34	0.023
non-IgG	34	23	
Type of free light chain			
kappa	32	32	0.848
lambda	22	25	
ISS			
stage 1, 2	40	36	0.483
stage 3	10	13	
unknown	4	8	
eGFR			
≥ 60mL/min	19	27	0.434
< 60mL/min	31	30	
unknown	4	0	
serum LDH level			
Mean serum LDH level	217.9 ± 95.5	240.0 ± 153.4	0.365
≥ 230U/L	17	22	0.551
< 230U/L	37	35	
High risk cytogenetic abnormality			
yes	12	14	0.999
no	24	27	
unknown	18	16	
Treatment line of DRd			
first line	22	18	0.331
second line or later	32	39	
High RDI of daratumumab			
yes	31	18	0.008
no	23	39	

Low RDI of dexamethasone			
yes	23	20	0.442
no	31	37	
Proteasome inhibitor exposure			
yes	32	34	0.06
no	0	5	
Proteasome inhibitor refractory			
yes	5	7	0.999
no	27	32	
Lenalidomide exposure			
yes	22	18	0.092
no	10	21	
Lenalidomide refractory			
yes	15	16	0.639
no	17	23	
ASCT prehistory			
yes	9	6	0.247
no	23	33	

RDI, relative dose intensity; DARA, daratumumab; LEN, lenalidomide; DEX, dexamethasone; ISS, international staging system; eGFR, estimated glomerular filtration rate; LDH, lactate dehydrogenase; DRd, daratumumab, lenalidomide plus dexamethasone; PI, proteasome inhibitors; IMiDs, immunomoduratory drugs; ASCT, autologous stem cell transplantation; NA, not available.

Supplementary Table S2. Patient Characteristics for high and low dexamethasone.

	**high RDI of DEX (n=43)**	**low RDI of DEX (n=68)**	***p* Value**
Age			
Mean age	76 ± 6	72 ± 7	0.005
≥75year	31	23	<0.001
<75year	12	45	
Sex			
male	25	38	0.846
female	18	30	
ECOG performance status			
0,1	9	14	0.999
2,3,4	34	54	
Tyep of monoclonal protein			
IgG	20	34	0.846
non-IgG	23	34	
Type of free light chain			
kappa	30	34	0.050
lambda	13	34	
ISS			

stage 1, 2	30	46	0.810
stage 3	10	13	
unknown	3	9	
eGFR			
≥ 60mL/min	18	28	0.999
< 60mL/min	23	38	
unknown	2	2	
serum LDH level			
Mean serum LDH level	240.9 ± 99.6	221.9 ± 144.0	0.449
≥ 230U/L	18	21	0.308
< 230U/L	25	47	
High risk cytogenetic abnormality			
yes	9	17	0.805
no	20	31	
unknown	14	20	
Treatment line of DRd			
first line	17	23	0.550
second line or later	26	45	
High RDI of daratumumab			
yes	21	28	0.441
no	22	40	
High RDI of lenalidomide			
yes	23	31	0.442
no	20	37	
Proteasome inhibitor exposure			
yes	25	41	0.646
no	1	4	
Proteasome inhibitor refractory			
yes	24	35	0.189
no	2	10	
Lenalidomide exposure			
yes	12	28	0.221
no	14	17	
Lenalidomide refractory			
yes	9	22	0.322
no	17	23	
ASCT prehistory			
yes	2	13	0.040
no	24	32	

RDI, relative dose intensity; DARA, daratumumab; LEN, lenalidomide; DEX, dexamethasone; ISS, international staging system; eGFR, estimated glomerular filtration rate; LDH, lactate dehydrogenase; DRd, daratumumab, lenalidomide plus dexamethasone; PI, proteasome inhibitors; IMiDs, immunomoduratory drugs; ASCT, autologous stem cell transplantation; NA, not available.
